# Neural correlates and predictors of speech and language development in infants at elevated likelihood for autism: a systematic review

**DOI:** 10.3389/fnhum.2023.1211676

**Published:** 2023-08-17

**Authors:** Jessica Morrel, Kripi Singapuri, Rebecca J. Landa, Rachel Reetzke

**Affiliations:** ^1^Center for Autism and Related Disorders, Kennedy Krieger Institute, Baltimore, MD, United States; ^2^Center for Neurodevelopmental and Imaging Research, Kennedy Krieger Institute, Baltimore, MD, United States; ^3^Department of Psychiatry and Behavioral Sciences, The Johns Hopkins University School of Medicine, Baltimore, MD, United States

**Keywords:** speech, language, neuroimaging, autism, infancy, neurophysiology, early development

## Abstract

Autism spectrum disorder (ASD) is an increasingly prevalent and heterogeneous neurodevelopmental condition, characterized by social communicative differences, and a combination of repetitive behaviors, focused interests, and sensory sensitivities. Early speech and language delays are characteristic of young autistic children and are one of the first concerns reported by parents; often before their child’s second birthday. Elucidating the neural mechanisms underlying these delays has the potential to improve early detection and intervention efforts. To fill this gap, this systematic review aimed to synthesize evidence on early neurobiological correlates and predictors of speech and language development across different neuroimaging modalities in infants with and without a family history of autism [at an elevated (EL infants) and low likelihood (LL infants) for developing autism, respectively]. A comprehensive, systematic review identified 24 peer-reviewed articles published between 2012 and 2023, utilizing structural magnetic resonance imaging (MRI; *n* = 2), functional MRI (fMRI; *n* = 4), functional near-infrared spectroscopy (fNIRS; *n* = 4), and electroencephalography (EEG; *n* = 14). Three main themes in results emerged: compared to LL infants, EL infants exhibited (1) atypical language-related neural lateralization; (2) alterations in structural and functional connectivity; and (3) mixed profiles of neural sensitivity to speech and non-speech stimuli, with some differences detected as early as 6 weeks of age. These findings suggest that neuroimaging techniques may be sensitive to early indicators of speech and language delays well before overt behavioral delays emerge. Future research should aim to harmonize experimental paradigms both within and across neuroimaging modalities and additionally address the feasibility, acceptability, and scalability of implementing such methodologies in non-academic, community-based settings.

## 1. Introduction

Autism spectrum disorder (ASD) is one of the most common and heterogeneous neurodevelopmental conditions, characterized by social communicative differences and the presence of focused interests repetitive behaviors, and sensory sensitivities (American Psychiatric Association [APA], 2013). ASD has an early-onset and increasing prevalence (1 in 36; Maenner, 2023). Delays in speech and language processing and development are hallmark characteristics of young autistic children, and one of parents’ first concerns, often reported before their child’s second birthday (Herlihy et al., 2015; Talbott et al., 2015). Moreover, language abilities are one of the strongest predictors of later academic performance, social relationships, and general quality of life (Petersen et al., 2013; Howlin and Magiati, 2017); however, the neurobiology underpinning the heterogeneity of speech and language development observed across the autism spectrum remains unclear. Elucidation of these neural mechanisms has the potential to enhance the lives of young children on the autism spectrum by accelerating early detection efforts, and by identifying salient developmental windows and mechanistic targets for early intervention.

Different neuroimaging techniques have been utilized to investigate the neurobiology of speech and language development in autism, each offering their own unique advantages and disadvantages. Structural techniques, such as magnetic resonance imaging (MRI), voxel-based morphometry (VBM), and diffusion tensor imaging (DTI), have shed light on the morphological changes and structural connectivity patterns in language-related brain regions. For example, a recent meta-analysis of 33 DTI studies reported that ASD participants, relative to typically developing (TD) peers, exhibited significantly lower fractional anisotropy (FA) across language-related white matter tracts, with deviations in language-related connectivity observed to be more pronounced in the left hemisphere relative to the right, and in children relative to adults (Li et al., 2022). Conversely, functional techniques, including functional magnetic resonance imaging (fMRI), functional near-infrared spectroscopy (fNIRS), and electroencephalography (EEG), have elucidated the dynamic neural activity underpinning speech and language processing, particularly regarding early cortical lateralization of language in the left hemisphere in neurotypically developing children and atypical language-related lateralization in young autistic children (Knaus et al., 2010). Together, these approaches have contributed to a deeper understanding of the complex neural mechanisms that support speech and language function as it emerges during early development, and how such neural mechanisms may diverge in autistic children. This review aims to synthesize evidence of the neurobiology of speech and language processing and development in infants at elevated likelihood for autism (EL infants). EL infants often share endophenotypes or subtle traits intermediate to genetic risk for and clinical manifestation of autism (Landa et al., 2007) with 20% of EL infants going on to receive a diagnosis of autism themselves (Ozonoff et al., 2011). Thus, prospective investigations focused on EL infants provide a unique opportunity to follow infants early in development, prior to the emergence of overt behavioral symptoms. Indeed, infants later diagnosed with autism have been found to exhibit differences in brain development within the first year of life, well before defining behavioral features fully emerge (Emerson et al., 2017; Hazlett et al., 2017).

While several reviews have examined neurobiological correlates of speech and language disorders present in autism, to our knowledge, no review has specifically examined the extent to which neuroimaging modalities have identified these differences in infant siblings of autistic children (at elevated likelihood or familial risk for developing autism; EL infants). Several reviews have focused on neuroimaging in ASD without a specific focus on speech or language (Ecker et al., 2015; Hernandez et al., 2015; Zhang and Roeyers, 2019). Others have focused on speech and language processing differences as it relates to ASD, though these papers focus on older children with an autism diagnosis, rather than exclusively on infants at elevated likelihood of developing autism (Schwartz et al., 2018; Chen et al., 2020; Key and D’Ambrose Slaboch, 2021; Cermak et al., 2022). Finally, although two reviews to date have examined the utility of neuroimaging techniques to describe and diagnose ASD in EL infants/toddlers (Ayoub et al., 2022; Clairmont et al., 2022), there has not been a systematic review that has specifically focused on early neural correlates and predictors of speech and language processing/development. The current review aims to fill this gap by asking the following research questions:

(1) What neuroimaging techniques have been used to understand the neurobiological mechanisms of speech and language function and development in infants at an elevated likelihood for ASD?
(2) What early neurobiological correlates and predictors of speech and language function and development have been identified across different types of neuroimaging modalities?

## 2. Materials and methods

The present study utilized the method of a systematic review as outlined by the Preferred Reporting Items for Systematic Reviews and Meta-Analyses (PRISMA) framework (Page et al., 2021).

### 2.1. Inclusion and exclusion criteria

Two authors independently conducted searches of the following databases in January 2023: PubMed, SCOPUS, and EBSCOhost (which included Academic Search Ultimate, APA PsyArticles, APA PsyInfo, ERIC, and MEDLINE). Search terms were chosen to identify primary research articles utilizing neuroimaging methods to assess neural correlates and predictors of speech and language in infants at elevated likelihood of developing autism (see Table 1). Papers were included in the initial search if they: (1) included at least one search term from each category; (2) were peer-reviewed journal articles; (3) were published in English; and (4) were published between 01/01/2012-01/18/2023. The date range was chosen to reflect the latest autism diagnostic criteria (American Psychiatric Association [APA], 2013).

**TABLE 1:** Search terms.

Diagnostic terms	Age-related terms	Outcome terms	Neuroimaging terms
Autism ASD Autistic Autism spectrum disorder	Infant sibling Infant Elevated likelihood Family history Familial history Risk	Language Speech Voice Linguistic	Neuroimaging Brain imaging Diagnostic imaging Magnetic resonance imaging MRI, fMRI, DTI Electroencephalography EEG, CAEP, AEP, ERP Magnetoencephalography MEG Functional near-infrared spectroscopy fNIRS

Papers were excluded from the current review if they were: (1) book chapters, monographs, and commentaries; (2) qualitative studies; or (3) studies that did not include a between-group comparison on a neural metric reflecting speech/language processing or development.

### 2.2. Study selection

The first and second authors conducted two independent and identical searches. Following the initial searches, results were exported for duplicate removal and cross-checked to ensure inter-rater reliability.

Following duplicate removal, the titles and abstracts of the remaining papers were independently screened by each author to determine eligibility using the following inclusion criteria:

1. Research design: at least one neuroimaging technique implemented in a cohort or case-control study and at least one neural metric of speech or language processing or examined a neural correlate or predictor (i.e., resting state metric) of a behavioral speech or language measure.
2. Participant groups: at least one cohort of participants diagnosed with autism spectrum disorder (ASD) or categorized as being at elevated likelihood for developing ASD (i.e., at least one older sibling with a diagnosis of ASD).
3. The average age at the time of neuroimaging: at least one neuroimaging measure completed in infancy (prior to or at 12 months of age).

**FIGURE 1:**
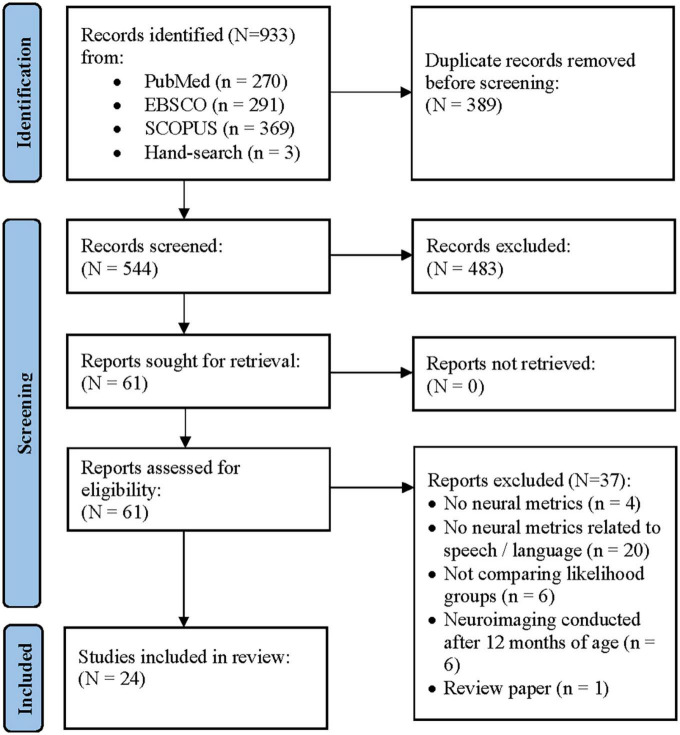
Preferred reporting items in systematic reviews and meta-analyses (PRISMA) flow diagram of the study selection procedure (Page et al., 2021).

Figure 1 shows a PRISMA flow diagram of the systematic review process. The initial search resulted in 930 papers. An additional three papers were identified through hand-search. Of the 544 records screened after duplicate removal, there were four disagreements (99.3% agreement). The two authors met to discuss these papers and were able to reach 100% consensus. Following the independent full-text review of the remaining 61 papers, the authors maintained 100% agreement on their final list of papers to be included in the systematic review. A total of 37 papers were excluded (see Supplementary Table 1 for details regarding why papers were excluded).

The authors extracted the following data from each included study, as it was available (see Table 2): study design, participant group sizes (% male), mean and SD chronological age of participants in each group, number of participants excluded from analyses, characteristics that groups were matched on, neuroimaging technique, and experimental paradigm. In addition, neural and behavioral measures related to speech and language were included. Finally, the authors included all significant between-group differences as well as significant brain-behavior relationships related to speech and language development in infants at elevated and low likelihood for ASD. All results were cross-examined by the senior author.

**TABLE 2:** Characteristics of included studies.

References	Analytic sample								
	EL infants		LL infants		Participants excluded	Characteristics matched on	Study design	Neuroimaging modality	Experimental paradigm
	N (% boys)	CA	N (% boys)	CA					
Swanson et al., 2017	EL ASD: 46 (77%) EL LD: 29 (65%) EL neg: 189 (54%)	EL ASD: 12.68 (0.69) months EL LD: 12.69 (0.62) months EL neg: 12.56 (0.62) months	104 (58%)	12.64 (0.74) months	EL: 118 LL: 39	Race	Cohort overall, cross-sectional brain	MRI	During natural sleep
Liu et al., 2019	19 (53%)	6.43 (1.27) weeks	15 (73%)	6.40 (1.03) weeks	EL: 1 LL: 6	age, sex, race, and family income	Cohort overall, cross-sectional brain	MRI	During natural sleep
Blasi et al., 2015	15 (66.67%)	147 (25) days	18 (38.89%)	154 (26) days	EL: 0 LL: 3	age and Mullen ELC	Cross-sectional study	fMRI	During natural sleep listening to three adult non-speech vocalizations: 1. emotionally neutral 2. emotionally positive 3. emotionally negative
Liu et al., 2020	1.5 months: 33 (57.58%) 9 months: 38 (60.52%)	1.5 (0.29) months 9.23 (0.38) months	1.5 months: 32 (59.38%) 9-months: 22 (50%)	1.55 (0.24) months 9.16 (0.37)	1.5 months: EL: 8 LL: 1 9 months: EL: 10 LL: 8	Age, sex, and race	Cohort Study	fMRI	Resting State during natural sleep
Liu et al., 2021	27 (70.37%)	9.19 (0.28) months	16 (43.75%)	9.13 (0.40) months	EL: 9 LL: 12	age, gender, race, family income, maternal education, amount of English exposure	Cohort Overall, Cross-sectional Brain	fMRI	Resting State during natural sleep while passively listening to 3 streams of nonsense speech: 1. Stressed Language 2. Unstressed Language 3. Random Syllables
Okada et al., 2022	Total Delayed = 18 (83.33%) EL = 16; LL = 2 Total Late-blooming = 25 (48%) EL = 18; LL = 7	9 months	Total typically developing = 23 EL = 11; LL = 12	9 months		age and motion	Cohort Overall, Cross-sectional Brain	fMRI	Resting State
Lloyd-Fox et al., 2013	18 (44.44%)	149.56 (26.75) days	16 (62.50%)	153.81 (25.67) days	EL: 11 LL: 9	Age, gender, developmental stage, looking time measures and motion artifact detected in fNIRS signal.	Cross-sectional study	fNIRS	Hemodynamic changes to three 3 conditions: 1. Visual social (silent; V-S) 2. Auditory vocal (V) 3. Auditory non-vocal (N-V) Presented in same order in repeating loops of trials
Edwards et al., 2017	21 (61.90%)	3.58 (0.39) months	17 (58.82%)	3.62 (0.35) months	EL: 8 LL: 1	age, birth weight, SES, parental age at birth	Cross-sectional Study	fNIRS	Auditory Stimuli: repeating (ABB) and non-repeating (ABC) trisyllabic sequences
Lloyd-Fox et al., 2018	Total EL: 20 (50%) EL-ASD: 5 EL-noASD: 15	149.35 (27.28) days	16 (62.5%)	153.81 (25.67) days	EL: 17 LL: 7	age, gender, looking behavior during task, Mullen ELC	Cohort overall, cross-sectional brain	fNIRS	Audio-visual conditions: videos of women performing games with 3 conditions 1. visual social (silent) 2. auditory non-vocal (with visual social) 3. auditory vocal (with visual social)
Pecukonis et al., 2021	Total EL: 14 (50%) ELA+: 5 ELA−: 9	215.2 days (range 174.0–242.0)	18 (50%)	210.9 days (range 187.0–241.0)	EL: 9 LL: 17	Mullen VDQ scores, demo (household income, maternal education, sex, race, ethnicity)	Cohort overall, cross-sectional brain	fNIRS	Auditory stimuli presented (during 3-, 6-, 9-, and 12-month visits) during fNIRS task: ABB and ABC speech syllables block trial design.
Riva et al., 2022	21 (57.89%)	12.25 (0.41) months	19 (42.85%)	12.38 (0.24) months	EL: 12 LL: 7	Sex, age, gestational weeks, and SES	Cross-sectional study	EEG	McGurk paradigm Stimuli: (1) congruent syllable /PA/ (2) congruent syllable /KA/ (3) incongruent fusion condition (4) incongruent mismatch condition
Seery et al., 2013	6 months: 29 (44.82%) 9 months: 45 (55.55%) 12 months: 43 (48.83%) N (full data at all three ages) = 14 (50.00%)	189.2 (9.6) days 278.1 days (8.5) 374.5 days (10.2)	6 months: 30 (50.00%) 9 months: 32 (40.65%) 12 months: 27 (40.74%) N (full data at all ages) = 12 (41.66%)	189.8 (11.4) days 277.8 days (6.9) 371.8 days (9.8)	T1: EL: 12 LL: 9 T2: EL: 3 LL: 5 T3: EL: 13 LL: 9	Sex	Cohort study	EEG	Audio stimuli: double-oddball paradigm: three consonant-vowel stimuli: (1) the standard: a voiced, unaspirated, retroflex stop (/a/) (2) native deviant: a voiceless, aspirated retroflex palatal stop (/ta/) (3) non-native deviant: voiced, unaspirated dental stop (/da/)
Arslan et al., 2020	25 (60%)	10-month: 10.32 (0.48) months 14-months: 14.28 (0.40) months	26 (65.4%)	10-months: 10.11 (0.37) months 14-months: 14.44 (0.68) months	EL: 9 LL: 9	Not reported	Cohort study	EEG	Audio-visual conditions; auditory stimuli = own name and unfamiliar name; visual stimuli = colored toys
Riva et al., 2018	EL-ASD: 20 (55%) EL-LI: 19 (57.89%)	EL-ASD: 12.36 (0.41) months EL-LI: 12.50 (0.49) months	TD: 22 (36.36%)	TD: 12.40 (0.25) months	Not reported	Sex, age, gestational weeks and socio-economic status (SES)	Cohort overall, cross-sectional brain	EEG	Auditory stimuli: non-speech oddball paradigm using 3 types of stimuli (1) standard tone-pairs (STD) (2) deviant for frequency (DEVF) (3) deviant for duration (DEVD)
Levin et al., 2017	25 (56%)	3.41 (0.49) months	14 (64.29%)	3.68 (0.44) months	EL: 4 LL: 5	sex and age	Cohort overall, cross-sectional brain	EEG	Resting state
Seery et al., 2014	35 (51.42%)	280.7 (10.2) days (Mean and SD based on total *n* = 40 before exclusion)	45 (51.11%)	281.3 (10.8) days	EL: 37 LL: 37	Age, sex, race, ethnicity, family income, and maternal education.	Cohort overall, cross-sectional brain	EEG	Audio stimuli: consonant-vowel stimuli using a double oddball paradigm.
Tran et al., 2021	Total EL: 36 (63.89%) ASD-concern: 14 (12 EL, 2 LL)	3 months (Mean and SD not reported)	Total LL: 27 (62.96%) No ASD-concern: 49 (24 EL, 25 LL)	3 months (Mean and SD not reported)	EL: 1 LL: 1	Family income	Cohort overall, cross-sectional brain	EEG	Auditory statistical learning (ASL) EEG paradigm. Infants exposed to continuous stream of concatenated syllables consisting of 4 different trisyllabic pseudo-words
Righi et al., 2014	6 months: 22 (not available ) 12-month: 25 (not available)	6 months (average not available) 12-month (not available)	6 month: 25 (not available) 12-month: 19	6 months (average not available) 12-month	6 months: EL: 6 LL: 2 12 month: EL: 3 LL: 7	Infant’s birth weight, parent’s age at birth, parent’s education level, family income	Cohort study	EEG	Audio stimuli with three consonant-vowel pairs: (1) standard condition (2) deviant Native Condition (3) deviant non-native condition.
Kolesnik et al., 2019	EL-ASD: 14 (88.24%) EL-Atyp: 21 (65.5%) EL-TD: 45 (43.75%)	EL-ASD: 8.83 (0.82) months EL-Atyp: 8.9 (0.69) months EL-TD 9.10 (0.84) months	14 (51.85)	9.29 (0.84) months	EL: 37 LL: 13	Not reported	Cohort study	EEG	Non-linguistic auditory oddball
Finch et al., 2017	EL-noASD: 39 (46.15%) EL-ASD: 13 (46.15%)	EL-noASD: 375.03 (8.9) days EL-ASD: 376.00 (14.3) days	44 (45.45%)	373.18 (9.1) days	EL-noASD: 28 EL-ASD: 10 LL: 29	Not reported	Cohort overall, cross-sectional brain	EEG	ERPs to stream of 3 different consonant-vowel stimuli presented in double-oddball procedure: 1. standard (80% of time; voiced, unaspirated, retroflex stop;/a/) 2. native deviant (10% of time; voiceless, aspirated retroflex palatal stop;/ta/) 3. non-native deviant (10% of time; a voiced, unaspirated dental stop;/da/).
Menn et al., 2022	EEG: 10 months: 8 (37.50%) 14 months: 14 (42.86%)	EEG: 10.26 (0.72) months 14.06 (0.5) months	EEG : 10 months: 10 (60%) 14 m: 9 (55.56%)	EEG: 10 (0.6) months 14.45 (0.6) months	8 months: *n* = 56 14 months: 51	Not reported	Cohort study	EEG	Presentation of video recording made of 5 sung nursery rhymes.
Peck et al., 2021	6 months: EL-ASD 6 months: 14 (57.14%) EL-noASD: 40 (47.50%) 12 months: Additional EL-ASD infants: 27 (62.96%)	6-months	N/A	N/A	Not reported	Sex, ethnicity, mean household income.	Cohort study	EEG	Audio stimuli: subset of an oddball phoneme speech task was used for analysis: only data recorded during the standard English phoneme (a voiced, unaspirated dental/da/).
Wilkinson et al., 2020	ELnoASD: 51 (49.02%) EL-ASD: 21 (66.67%)	3, 6, 9, 12, 18, 24 m visits–mean and SD at each visit not reported	58 (51.72%)	3, 6, 9, 12, 18, 24 m visits–mean and SD at each visit not reported	EL = 51 LL = 39	Sex, paternal education, household income, race, ethnicity.	Cohort study	EEG	Resting state
Hedenius et al., 2022	49 (44.90%)	5 months at EEG; mean and SD only available for MSEL age	18 (50%)	5 months at EEG; mean and SD only available for MSEL age	EL: 1 LL: 5	Not reported	Cohort overall, cross-sectional brain	EEG	Steady state visually evoked potentials (visual paradigm of 2 blocks, motion or form)

ASD, autism spectrum disorder; TD, typically developing; LD, language delay; CA, chronological age; ELC, early learning composite; SD, standard deviation; EL, elevated likelihood; LL, low likelihood; MRI, magnetic resonance imaging; fMRI, functional magnetic resonance imaging; EEG, electroencephalography; fNIRS, functional near-infrared spectroscopy; SES, socioeconomic status.

### 2.3. Assessment of risk of bias

This review paper appears to have a low risk of bias overall. The reviewers took all steps to minimize bias. The authors clearly describe the methods used to provide an overview of the literature on the topic of interest. The search terms were defined prior to the start of the process and were uniformly applied to all four databases. Additionally, the inclusion and exclusion criteria were clearly stated and followed as closely as possible. A formal quality assessment of the studies was not conducted during the exclusion process. Two reviewers achieved consensus on all included articles. Taken together, the methods described in this review were closely followed, and no deviations occurred to the knowledge of the reviewers.

## 3. Results

### 3.1. Study characteristics

Twenty-four studies met the criteria for inclusion in this review (see Table 2 for study characteristics). The included studies were conducted across the world, including in the United Kingdom, Italy, Belgium, Sweden, and Netherlands, as well as several states throughout the United States. Together, these studies collected neuroimaging data from participants aged 6 weeks to 12 months, as well as behavioral data collected between the ages of 4- and 36-months. All included studies implemented either a cohort (*n* = 20) or cross-sectional study (*n* = 4) study design. Of the 20 cohort studies, 12 were cohort overall, but cross-sectional for neuroimaging data, due to missingness of neural data. The studies included two main participant groups—those with an older sibling with a confirmed diagnosis of ASD (EL infants), and those without (LL infants). Of the prospective studies, 10 used later results on developmental and autism-specific diagnostic instruments to further subdivide the original participant groups (an example of a potential participant subdividing is LL infants, EL infants-No ASD, EL infants-ASD).

Final analytic sample sizes ranged from 8 to 264 for EL infants and 9 to 104 for LL infants, with a range of 2 to 157 total participants excluded. Based on information from 22 of the 24 papers, we found that infant participants were excluded from the final analytic sample due to reasons captured by five common themes: (1) incomplete or poor neuroimaging data due to infant waking up during natural sleep paradigms or excessive fussiness (Seery et al., 2013, 2014; Edwards et al., 2017; Levin et al., 2017; Lloyd-Fox et al., 2018; Kolesnik et al., 2019; Liu et al., 2019, 2020; Arslan et al., 2020; Tran et al., 2021; Menn et al., 2022; Riva et al., 2022); (2) equipment or experimental failure (Seery et al., 2014; Lloyd-Fox et al., 2018; Pecukonis et al., 2021); (3) not enough data points due to attrition (Pecukonis et al., 2021); (4) prior exposure to the language stimuli (Seery et al., 2013, 2014); and (5) brain or perceptual abnormalities (Hedenius et al., 2022). One study included participants from a larger study and did not have information on the excluded participants (Riva et al., 2018). One study did not include group breakdown of participants excluded, but 56 infants at 10 months, and 52 at 14 months were excluded due to excessive movement, noisy channels, bad neighboring channels, few trials after artifact rejection, wrong testing age, and better data being available at a different time point (Menn et al., 2022). Of all participants excluded across studies, there appeared to be no significant difference in the proportion of EL vs. LL infants who were excluded; on average 27.44% of EL infants and 27.14% of LL infants were excluded.

Several different behavioral measures were included across studies to assay language development. Both clinician-administered measures, such as the Mullen Scales of Early Learning (MSEL; Mullen, 1995); and parent-report measures like the Vineland Adaptive Behavior Scales (VABS; Sparrow et al., 2016) and MacArthur-Bates Communicative Development Inventories (MB-CDI; Fenson et al., 2007), were leveraged to assess the emergence of language and communication at specific developmental time points.

### 3.2. Synthesis of results

Neuroimaging approaches included MRI (*n* = 2), fMRI (*n* = 4), fNIRS (*n* = 4), and EEG (*n* = 14). Although magnetoencephalography (MEG) is a common neuroimaging technique that has been previously implemented to examine the neurobiology underlying speech and language acquisition in infancy, our systematic review did not yield any studies using this neuroimaging technique to assay the neurobiology of speech and language processing or development in EL vs. LL infants. Here we summarize the 24 included studies by neuroimaging technique implemented.

#### 3.2.1. Structural studies

Two MRI studies scanned infants during natural sleep. One study included gray matter findings (Swanson et al., 2017) while the other examined white matter tracts (Liu et al., 2019).

One novel investigation examined associations between volumes of subcortical structures, including the amygdala, thalamus, and caudate at age 12 months, and later language skills. EL infants that went on to receive an autism diagnosis (EL-ASD) exhibited differing associations between thalamus and amygdala volumes at age 12 months and verbal language ability at 24 months {as measured by the MSEL verbal developmental quotient [MSEL (VDQ)]}, compared to EL infants who exhibited language delays (EL-LD). Additionally, the EL-ASD and EL-LD groups exhibited different associations between the thalamus, amygdala, and caudate volumes and a measure of receptive language. While identical brain-behavior phenotypes were observed in ASD infants with and without language delay, they were distinct from those of EL-LD infants (Swanson et al., 2017), suggesting autism-specific neurobiology and the potential of a heritable or familial biological trait that does not always lead to behavioral expression of autism. Fractional anisotropy (FA) within two dorsal white matter tracts—the superior longitudinal fasciculus (SLF) and arcuate fasciculus (AF)—was examined to measure the laterality and structural connectivity of major language regions. Liu et al. (2019) found that LL infants exhibited significantly higher mean FA in the left SLF compared to EL infants. In contrast, EL infants showed significantly higher FA in the right SLF than LL infants, suggesting a more rightward lateralization than LL infants in the SLF. Finally, higher FA in the left AF and left SLF at 6 weeks was associated with a measure of receptive language at 18 months of age (Liu et al., 2019).

#### 3.2.2. Functional studies

##### 3.2.2.1. Functional magnetic resonance imaging

Four fMRI studies were included and reviewed. Two fMRI studies assessed resting-state functional connectivity (rs-fcMRI; Liu et al., 2020; Okada et al., 2022), while the remaining two studies utilized passive listening paradigms (Blasi et al., 2015; Liu et al., 2021). All identified fMRI studies scanned infants during natural sleep. Investigations utilizing rs-fcMRI and DTI metrics found that EL infants exhibited alterations in white matter tracts connecting the frontal, temporal, and subcortical regions of the brain—functional connectivity implicated in language development and function. Such differences were identified within the first year of life (Liu et al., 2020; Okada et al., 2022). For example, one study found that compared to LL infants, EL infants as young as age 6 weeks showed altered functional connectivity between temporal [i.e., left Heschl’s gyrus (HG), posterior superior temporal gyrus (pSTG)] and somatosensory regions thought to subserve auditory-motor integration. In addition, EL infants showed limited development of long-range functional connectivity between these regions from 6 weeks to 9 months of age (Liu et al., 2020).

Another study utilized a data-driven clustering approach to categorize EL and LL infants’ developmental trajectories of receptive language (as measured by the MSEL; Mullen, 1995) from age 6 to 36 months (typical = trajectory consistently falling in the upper end of the normative range; late-blooming = 6 to the 18-month trajectory in the lower end of the normative range, with gains noted between age 18 and 36 months; and developmental delay = overall delayed trajectory from age 6 to 18 months). Infants who were categorized as typical and late-blooming showed stronger cerebro-cerebellar connectivity between the right Crus I and all ROIs examined (frontal cortex, supplementary motor area, basal ganglia, and thalamus) compared to infants in the developmental delay group. There were no significant differences observed between typical and late-blooming language cohorts (Okada et al., 2022). Two studies scanned infants during natural sleep, passive-listening paradigms. In one study, Blasi et al. (2015) investigated neural responses to human vocalizations in sleeping infants aged 4–7 months, to assay differences in preference for human voice processing, sensitivity to affect, and the influence of parent-child interaction on brain responsivity. The infants were exposed to three categories of adult non-speech vocalizations (emotionally neutral, such as yawning, sneezing, or coughing; emotionally positive, such as laughter; and emotionally negative, such as crying) and familiar non-voice environmental sounds (e.g., toys and running water). The results demonstrated that LL infants (*n* = 18) showed an early preference for human voice processing, with stronger activation in the middle and superior temporal regions of the brain and the medial frontal gyrus compared to EL infants (*n* = 15). Additionally, LL infants exhibited a more pronounced response to sad voices in the right fusiform gyrus and left hippocampus than EL infants. Finally, the relationship between risk status and infant processing of voice in the right medial frontal gyrus was influenced by infant behavior, characterized by active engagement during observed mother-infant interactions. This suggests that group differences in neural responsiveness may be partially explained by variations in infant-driven social experiences (Blasi et al., 2015).

Another study (Liu et al., 2021) quantified infant neural responses to three counterbalanced speech stream exposure fMRI paradigms. In brief, this paradigm included one condition with stressed language, which included both statistical and prosodic cues for word boundaries; one condition with unstressed language, which included statistical cues only; and one random syllable condition, as a control. During the stressed language condition, LL infants showed significantly greater activation in the left amygdala than EL infants. The LL infants exhibited greater signal increases to stressed vs. unstressed language in the left temporal regions involved in language processing (the left STG and HG), as well as increased functional connectivity between the bilateral HG and right anterior insula across exposure to the stressed language condition. Across groups, greater signal increases in the left STG/HG at 9 months were associated with higher expressive language scores at 36 months. For EL infants, greater signal increases at 9 months were associated with lower levels of ASD symptomology at 36 months (Liu et al., 2021).

##### 3.2.2.2. Functional near-infrared spectroscopy

Four fNIRS studies investigated hemodynamic responses to different auditory stimuli in infants (Lloyd-Fox et al., 2013, 2018; Edwards et al., 2017; Pecukonis et al., 2021).

Across two different studies, one group examined hemodynamic changes to three audiovisual conditions [visual social (V-S), auditory vocal (V), and auditory non-vocal (N-V)] at 4–6 months of age (Lloyd-Fox et al., 2013) and their associations with autism outcomes at 36 months (Lloyd-Fox et al., 2018). Specifically, Lloyd-Fox et al. (2013) reported that LL infants demonstrated significantly greater hemodynamic response over the anterior superior temporal sulcus (aSTS) region—suggested to play a role in social perception—to the vocal vs. non-vocal condition (). At 36 months, the participants were assessed for autism using the autism diagnostic observation schedule (ADOS-2; Lord et al., 2012) and the autism diagnostic interview (ADI-R; Rutter et al., 2003). At this time point, LL infants and EL-noASD infants exhibited vocal selectivity in the left temporal region (aMTG-STG, pSTS-TPJ) as evidenced by stronger oxy-hemoglobin (oxyHb) responses to the vocal vs. non-vocal conditions (Lloyd-Fox et al., 2018).

Two additional investigations examined hemodynamic responses to repeating (ABB) and non-repeating (ABC) trisyllabic speech-like sequences to determine whether LL and EL infants differ in their ability to discriminate repetitive vs. random speech-like stimuli (Edwards et al., 2017; Pecukonis et al., 2021). Only one of the studies sought to determine whether EL infants show altered exposure-dependent changes to repetitive vs. random speech-like stimuli (Edwards et al., 2017). Edwards et al. (2017) found that female LL infants showed lower oxyHb responses to the last 4 ABB trials than the first 4 ABB trials, and their oxyHb responses to the last 4 ABB trials were significantly lower than EL females; suggesting that LL females may habituate to speech over time, more than EL females. In addition, EL female infants exhibited significantly higher oxyHb responses to auditory stimuli in the left anterior region compared to EL male infants and both sex groups of LL infants (Edwards et al., 2017). Using the same paradigm, Pecukonis et al. (2021) found that LL infants showed greater oxyHb concentrations to both types of speech bilaterally in anterior regions (LL > EL) while EL infants showed relatively similar neural activity to speech across all regions of interest (ROIs). Finally, within the LL group, only, higher oxyHb concentration values in the left anterior region at 6 months significantly correlated with higher VDQ scores at 24 months (Pecukonis et al., 2021). Unlike Edwards et al. (2017) and Pecukonis et al. (2021) did not stratify results by sex.

##### 3.2.2.3. Electroencephalography

Fourteen EEG studies are described below. Of these studies, six utilized EEG time-domain metrics [i.e., various event-related potential (ERP) components], five utilized frequency-domain measures (e.g., EEG power and coherence), and three studies implemented novel metrics (e.g., machine learning) to examine speech and language processing differences in EL vs. LL infants.

###### 3.2.2.3.1. ERP components

Four EEG studies (Seery et al., 2013, 2014; Finch et al., 2017; Riva et al., 2018) examined differences in ERP components elicited through auditory oddball paradigms, where a series of repetitive, standard auditory stimuli are occasionally interrupted by infrequent, deviant sounds (odd balls; Näätänen et al., 2007). Another study implemented a classic McGurk effect paradigm to examine neural correlates of audiovisual integration among EL vs. LL infants (Riva et al., 2022). Finally, Arslan et al. (2020) examined differences in neural responses to infants’ hearing their own name vs. an unfamiliar name.

Three studies used the same oddball paradigm (from Rivera-Gaxiola et al., 2005) which consisted of a stream of three consonant-vowel syllables: a standard stimulus (voiced, unaspirated, retroflex stop;/?a/) 80% of the time, a native deviant stimulus (voiceless, aspirated retroflex palatal stop;/ta/) 10% of the time, and a second non-native deviant (voiced, unaspirated dental stop;/da/) 10% of the time (Seery et al., 2013, 2014; Finch et al., 2017). In Seery et al. (2013), ERP responses to speech stimuli were recorded at 6 and 12 months to examine whether perceptual narrowing (i.e., increased sensitivity for native language-specific speech processing and decreased sensitivity for non-native language-specific processing) may be predictive of later autism symptomology (language processing, in particular). The results of this study found that at 6-months the P150 amplitude (occurring 150–300 ms after the onset of the stimulus) was similar for both native and non-native stop-consonant contrasts compared to the standard/da/for both groups of infants. At 12 months, both groups of children (including those diagnosed with ASD at 36 months of age) ceased to show a difference in their P150 amplitude of the non-native contrast, relative to the standard, in support of the perceptual narrowing hypothesis. In contrast, significant group differences were found for lateralization of ERP responses to speech between 6 and 12 months. While the EL group showed no difference in their responses to the speech stimuli across hemispheres, the LL group exhibited a more negative response over the right hemisphere relative to the left (Seery et al., 2013).

Building on Seery et al. (2013)’s findings, Finch et al. (2017) examined whether group differences (non-ASD, EL-non-ASD, vs. EL-later ASD) in lateralization of ERPs to speech sounds exist at 12 months of age and whether these patterns related to later developmental profiles. At 12 months, the EL-non-ASD group showed a more positive later-going slow wave (LSW) laterality index in the left hemisphere, compared to the EL-later ASD group. When infants with lower language scores at 12 months were excluded from analyses, non-ASD participants also exhibited a more positive LSW mean amplitude over the left hemisphere, compared to ASD infants (Finch et al., 2017).

In contrast to Seery et al. (2013, 2014) and Finch et al. (2017) aimed to determine whether the P150 of EL infants differed from LL infants in response to the first, second, and third consecutive repetitions of a standard stimulus (as described above). Additionally, this study examined the relation between these 9-month-old responses and subsequent 18-month-old behavior, as well as examining infants’ responses to a deviant stimulus to understand whether any potential atypicality was specifically related to stimulus repetition or more generally linked to speech processing (Seery et al., 2014). The findings revealed that EL infants exhibited a larger P150 amplitude to repeated standard speech stimuli compared to LL infants in the frontal region; and in EL infants only, higher P150 amplitudes to the standard stimuli over the frontal and central regions were associated with higher expressive language scores (Seery et al., 2014).

Riva et al. (2018) examined the P3 to “deviant” stimuli and the mismatch response (MMR) between the deviant vs. standard stimuli. This study aimed to determine whether the P3/MMR components (1) differentiated the LL and EL groups and (2) related to later language development and autism symptomology at 20 months. EL- ASD infants exhibited an overall higher P3 amplitude compared to LL infants across stimulus types and hemispheres. EL-ASD infants and EL infants with later language impairment (EL-LI) had a significantly longer peak latency relative to the TD group. Twelve-month-olds’ peak latency of the mismatch response duration deviant (MMRD) was associated with expressive vocabulary scores at 20 months of age. That is, faster MMRD at 12 months was associated with more words produced at 20 months (Riva et al., 2018).

In a follow-up study, Riva et al. (2022) investigated differences in audiovisual (AV) integration between EL and LL infants using ERP responses within a McGurk paradigm, which demonstrates the interaction between auditory and visual perception in speech processing by presenting mismatched auditory and visual speech stimuli, leading to the perception of a fused syllable (McGurk and MacDonald, 1976). This paradigm included four AV stimuli: congruent PA, congruent KA, incongruent fusion, and incongruent mismatch. Mean ERP amplitudes were analyzed in the following time windows: 100–250 ms for the visual effect in the occipital area and 350–650 ms for AV stimuli in the frontal (left and right) and temporal areas (left and right). They found that, in the left temporal hemisphere, LL infants exhibited a higher mean amplitude for the mismatch condition compared to both congruent conditions, while EL infants showed no differences between ERP responses to congruent and incongruent stimuli. This suggests that the LL group demonstrated AV integration effects over the left temporal area with larger mean amplitude responses to the mismatch condition, whereas the EL group showed no difference in their ERP responses to congruent and incongruent AV stimuli. However, both groups showed no differences in the earlier stages of visual processing (occipital early negative response), indicating that the impairment in the EL group was specific to AV speech integration.

Finally, Arslan et al. (2020) investigated whether EL and LL infants exhibit differences in ERPs in response to their own names at 10 and 14 months of age. LL infants displayed an overall increased positive neural activity in the frontal regions, compared to EL infants. As compared to LL infants, EL infants exhibited significantly reduced positive-going activity in response to their own name at 14 months. In addition, only 14-month-old EL infants displayed reduced neural responses to hearing their own name vs. an unfamiliar name. Frontal positive responses and the difference scores in the frontal areas between hearing one’s own name and an unfamiliar name correlated with the receptive language scores at 10 months (Arslan et al., 2020).

###### 3.2.2.3.2. EEG power and coherence

Five EEG investigations (Righi et al., 2014; Levin et al., 2017; Kolesnik et al., 2019; Wilkinson et al., 2020; Tran et al., 2021) examined EEG measures of spectral power and/or phase coherence within frequency bands or trials (i.e., inter-trial coherence; Kolesnik et al., 2019).

Two studies examined EEG power in 3-month-old EL and LL infants: one in a resting state EEG paradigm (Levin et al., 2017), and another in a passive listening paradigm (Tran et al., 2021). In the study using a resting state EEG paradigm, EL infants at 3 months showed significantly lower power over the frontal region in the high-alpha and beta bands (Levin et al., 2017). Another study that scanned infants during passive exposure to a stream of trisyllabic pseudo-words, however, found that 3-month EEG power was not significantly different between infants with (ASD-concern) and without (no-ASD-concern) autism concerns at 18 months. In contrast, 3-month phase coherence differentiated these groups. Indeed, at 3 months, infants later categorized in the ASD-concern group [groups defined using the ADOS Toddler Module (ADOS-T; Luyster et al., 2009) calibrated severity score (CSS; Esler et al., 2015) ranging from 1 to 10 where CSS > 4 = ASD-concern and CSS < 4 = No-ASD-concern] showed reduced theta and alpha coherence at left frontal-central electrodes (F9-C3) as compared to infants in the no-ASD-concern group (Tran et al., 2021). Using a double oddball paradigm, a third study examined linear coherence in gamma frequency bands between frontal and temporoparietal regions at 6- and 12 months. While no neural differences were significant between likelihood groups at 6 months, at 12 months, LL infants displayed higher linear coherence than EL infants. LL infants without later ASD diagnosis displayed higher linear coherence compared to all EL infants. In addition, EL-noASD infants showed higher coherence compared to EL-ASD infants (Righi et al., 2014).

In another study (Kolesnik et al., 2019), eight-month-old EL and LL infants were tested in a non-linguistic auditory oddball paradigm. The paradigm consisted of a pure tone at 500 Hz, presented with a 77% probability (standard tone), and two infrequent deviant tones—a white noise deviant and a pure tone of 650 Hz (pitch deviant)—each presented with an 11.5% probability. Compared to EL infants with typical development, infants who later developed ASD showed decreased neural habituation, as evidenced by an increase in gamma activation and significantly greater 10–20 Hz inter-trial coherence for repeated tones.

Of the five reported EEG studies in this section, four reported brain-language associations. In one resting-state study, reduced high-alpha power in frontal regions at 3 months was associated with lower expressive language scores at 12 months (Levin et al., 2017). Consistent with these findings, across all outcome groups, Tran et al. (2021) found that left frontal-central alpha coherence at 3 months was correlated with word production scores at 18 months (as measured by the MB-CDI; Tran et al., 2021). In contrast to these findings, a resting-state EEG study found that EEG-language associations differed by ASD outcome in EL infants, such that higher estimated 6-month gamma power was correlated with increased 24-month language functioning in HR-NoASD infants, but decreased 24-month language functioning in HR-ASD infants (Wilkinson et al., 2020). Somewhat consistent with findings from Wilkinson et al. (2020), using a non-linguistic auditory oddball paradigm, Kolesnik et al. (2019) found that 8 month cortical reactivity scores (derived by averaging z-scores for the evoked gamma and inter-trial coherence responses) were associated with reduced growth in receptive language skills between 8 and 36 months of age in high risk infants. Taken together, the differences in patterns of brain-language associations across the studies summarized here may potentially reflect early dynamic neurodevelopmental changes. Indeed, the former two studies, although using different EEG paradigms, focused on 3 month infants, while the latter two EEG studies focused on relatively older infants aged 6 and 8 months, respectively. Future work is needed to determine whether the reported brain-language associations are stable during early development or change dynamically over time.

###### 3.2.2.3.3. Novel EEG paradigms

Three investigations used novel EEG paradigms and/or analytic approaches beyond looking explicitly at more traditional EEG time- or frequency-domain metrics.

Using non-linear language-related EEG measures collected during an auditory oddball paradigm, Peck et al. (2021) aimed to predict ASD diagnosis in 6- and 12-month-old infants. For this study, the paradigm from Seery et al. (2013) was implemented but only EEG data recorded to the standard English phoneme (voiced, unaspirated dental/da/) were used. Employing a combination of Pearson correlation feature selection and a support vector machine (SVM) classifier, 100% predictive diagnostic accuracy was achieved at both ages. However, the predictive features differed between the models trained on 6-month versus 12-month data. At 6 months, features were biased toward measures from central electrodes, power measures, and frequencies in the alpha range. In contrast, at 12 months, features were more evenly distributed between power and non-linear measures and biased toward frequencies in the beta range. Notably, the diagnostic prediction accuracy substantially decreased in a larger, more behaviorally heterogeneous 12-month sample, from 100% with the matched dataset to 7% with the full dataset, suggesting an inability to effectively separate the two diagnostic outcome classes after expanding the EL-ASD group.

Another study investigated the association between neural processing of continuous speech (i.e., speech tracking), language development, and later autism symptoms. Infants at 10- and 14-months-old listened to five, sung nursery rhymes while continuous EEG data were recorded. Speech-brain coherence to these nursery rhymes served as a measure of infants’ ability to align neural activity with ecologically relevant speech input, with an emphasis on stressed syllables, syllables, and phonemes. The association between speech-brain coherence at 10 and 14 months and receptive and expressive vocabulary scores at 24 months [as measured by the parent-report, MacArthur-Bates Communicative Development Inventories (MCDI)] and autism symptoms at 36 months (as measured by the ADOS) was also examined. Results revealed no significant difference in speech-brain coherence between the EL and LL groups or any association between speech-brain coherence and later autism symptoms. However, a significant association was found between speech-brain coherence and later vocabulary development, with higher coherence in the stressed syllable rate correlating with higher parent-reported receptive and expressive vocabulary. This association was more pronounced in the 10-month-old EL group and is suggested to reflect individual differences in infants’ word segmentation skills, which have been found to be critical for language acquisition.

Finally, Hedenius et al. (2022) moved beyond the auditory domain and investigated the associations between global motion processing (GMP) and global form processing (GFP) with language and motor skills in 5-month-old EL and LL infants, assessed again at 18 months. GMP involves the integration of local visual motion signals into a global perception of motion, while GFP focuses on form processing and object recognition (Hedenius et al., 2022). The study found a significant negative relation between GMP laterality at 5 months and receptive language subtest on the MSEL at 18 months. The association between GMP laterality and language skills was consistent regardless of the infant’s likelihood for autism status, suggesting that atypical lateralization of GMP may be associated with lower motor and language scores at 18 months. These findings highlight the role of GMP in the development of motor and language skills, with potential implications for autistic children who may show atypical lateralization and processing challenges.

## 4. Discussion

To our knowledge, this is the first systematic review to synthesize evidence from different neuroimaging techniques used to understand the mechanisms underlying speech and language function and development in EL infants (0 to 12 months). The main findings from this review can be categorized into three main themes, suggesting that EL infants exhibit: (1) atypical language-related lateralization; (2) alterations in structural and functional connectivity; and (3) mixed profiles of neural sensitivity to speech and non-speech sounds. While these general trends were observed across different neuroimaging modalities, variability in brain-language associations along with different experimental paradigms and task demands implemented across studies makes it difficult to draw strong conclusions based on the limited evidence available. We discuss the main trends observed as well as limitations and future directions below.

### 4.1. Main trends

#### 4.1.1. Atypical language-related lateralization

Patterns of language-related lateralization were inconsistent across studies. In line with findings in autistic children and adolescents (Knaus et al., 2010), three studies found evidence suggesting atypical leftward lateralization (i.e., language-related lateralization) in infants at elevated likelihood for autism (Seery et al., 2013; Finch et al., 2017; Liu et al., 2019), as early as 6 weeks of age (Liu et al., 2019). Only Liu et al. (2019) found that these alterations were associated with later receptive language outcomes. Two EEG investigations identified similar patterns of atypical language-related lateralization; one in EL infants between 6 and 12 months of age in EL infants (Seery et al., 2013), and another in EL infants at 12 months of age who did not go on to receive an autism diagnosis (Finch et al., 2017). In contrast to these findings, one fMRI study (Blasi et al., 2015) and one fNIRS study (Pecukonis et al., 2021) did not find significant differences in the laterality of functional responses to human non-speech and speech stimuli at 6-months of age. Future work using a combination of resting-state and awake passive listening paradigms should be implemented to clarify at which point in development the left-lateralization of speech and language processing becomes disrupted in EL infants and the extent to which this phenomenon is mediated by experimental tasks.

#### 4.1.2. Alterations in structural and functional connectivity

Altered structural and functional connectivity in the first year of life was consistently found across studies. However, specific white matter tracts and regions of interest differed across studies. With regards to structural connectivity, Liu et al. (2019) found that EL infants showed reduced FA in left hemisphere white matter tracts subserving language function. Altered functional connectivity was identified, as well. For example, Liu et al. (2020) and Okada et al. (2022) both found that EL infants exhibited alterations in white matter tracts in the first year of life. Liu et al. (2020) found altered functional connectivity between temporal and somatosensory regions in EL infants, while Okada et al. (2022) showed that infants with developmental delays had reduced cerebro-cerebellar connectivity than infants with typical and late-blooming language development. Additionally, Liu et al. (2021) found that, relative to LL infants, EL infants demonstrated reduced functional connectivity between the bilateral HG and the right anterior insula. In line with these findings, two EEG studies found that infants who went on to have concerns for or a diagnosis of autism showed reduced coherence in theta and alpha frequencies in the left frontal-central region (Tran et al., 2021), as well as in gamma frequency bands (Righi et al., 2014) during their first year of life. Taken together, the evidence provides support for a hypoconnectivity theory of autism, though the locations and frequency bands at which this reduced connectivity is found are mixed. While the majority of reviewed studies found that infant siblings of children with ASD exhibit atypical speech and language structural and functional connectivity in cortical brain regions that subserve language function, there is an emerging body of evidence that has highlighted subcortical structural and functional alterations in EL infants (Blasi et al., 2015; Swanson et al., 2017; Okada et al., 2022). Indeed, three studies included in this review found EL-infant structural or functional differences associated with the amygdala, thalamus (Okada et al., 2022), caudate (Swanson et al., 2017), cerebellum (right Crus I; Okada et al., 2022) and the hippocampus (Blasi et al., 2015). Future studies should continue mapping out the developmental trajectory of structural and functional connectivity related to speech and language processing from subcortical to cortical structures in EL infants. Such work has the potential to elucidate the earliest level of the brain at which neural structure and function related to language development may diverge from neurotypical development.

#### 4.1.3. Mixed profiles of neural sensitivity to human non-speech and speech stimuli

Varying response patterns to speech and non-speech stimuli were found across studies, with the majority of studies showing some pattern of reduced sensitivity (*n* = 6), and only four showing some pattern of elevated sensitivity. Results from two fMRI studies found that EL infants showed a reduced early preference for human voice processing between ages 4 and 7 months (Blasi et al., 2015), and reduced signal increases in left temporal regions during the stressed language condition at 9 months of age (Liu et al., 2021), compared to LL infants. In line with these fMRI findings, an ERP study found that EL infants exhibited a reduced neural response to their own name at 14 months (Arslan et al., 2020); and an fNIRS study (Pecukonis et al., 2021) found reduced neural responsivity to speech sounds in the bilateral anterior regions at age 6 months, which was found to be driven by infants later diagnosed with ASD. This pattern of reduced sensitivity was also found in an additional fNIRS study (Lloyd-Fox et al., 2013) and an ERP study (Riva et al., 2022) utilizing audiovisual stimuli in EL vs. LL infants at ages 6 and 12 months, respectively. From a mechanistic perspective, one study found that infants exhibiting smaller voltage differences between congruent and incongruent conditions tended to show increased hyporesponsiveness to sensory input, as measured by a parent-report sensory profile measure (Riva et al., 2022), suggesting that the reduced neural sensitivity may be related to a general hyporesponsive sensory processing profile. Taken together, these results suggest that some EL infants may have reduced neural sensitivity to a variety of speech and non-speech human vocalizations (e.g., speech syllables, child’s own name, and human non-speech vocalizations) as early as age 4 months (Blasi et al., 2015).

This pattern of reduced sensitivity to speech and non-speech sounds was not consistently found across studies. Indeed, two EEG studies revealed a pattern of heightened responses to speech stimuli in EL infants relative to LL infants (Seery et al., 2014; Riva et al., 2018). Seery et al. (2014) used repeated standard speech stimuli and a deviant stimulus to investigate the P150 component, while Riva et al. (2018) examined the P3 component in response to “deviant” stimuli and the mismatch response (MMR) between deviant and standard stimuli. The findings of Seery et al. (2014) and Riva et al. (2018) both indicated that EL infants exhibited atypical neural responses to speech stimuli, with larger P150 and P3 amplitudes, respectively. Riva et al. (2018) found a significant association between larger P3 amplitude and increased autism symptoms, suggesting that enhanced neural responses to auditory discrimination processing early in life might be specifically predictive of ASD symptomatology (Riva et al., 2018). In contrast, Seery et al. (2014) found heightened responsivity to speech stimuli was related to better language outcomes in EL infants only, suggesting that heightened neural responsivity may reflect a compensatory mechanism by which the infant is able to capitalize on to attend to incoming speech sounds. Consistent with these two studies, another EEG study using pure tones found that infants who later developed ASD showed increased gamma activation and greater 10–20 Hz inter-trial coherence for repeated tones (Kolesnik et al., 2019). Finally, somewhat aligned with the above an fNIRS study found that EL female infants exhibited significantly higher oxyHb responses to speech stimuli in the left anterior region compared to EL male infants and both sex groups of LL infants (Edwards et al., 2017). Taken together this body of evidence suggests that some EL infants may present with reduced repetition suppression or habituation of neural responses to repeated auditory stimuli, which may contribute to challenges encoding novel, complex speech sounds needed for successful language acquisition (Kolesnik et al., 2019).

Overall the findings from this body of evidence support the hypothesis that different patterns of speech and non-speech processing in EL infants may arise from a potentially dysregulated sensory profile, leading to hypo- or hyper-sensitivity to incoming auditory stimuli (Seery et al., 2014; Edwards et al., 2017; Riva et al., 2018; Pecukonis et al., 2021). The differences in patterns of findings across studies are consistent with previous literature in older children, adolescents, and adults on the autism spectrum (Jeste and Nelson, 2009; Schwartz et al., 2018; Chen et al., 2020; Key and D’Ambrose Slaboch, 2021). The studies reviewed herein contribute valuable insights into how these different sensory profiles may begin to emerge in infancy, prior to formal autism diagnoses, and, how they may relate to later speech and language outcomes. Further research is needed to disentangle the complex pattern of associations between sensory processing and language skills in EL infants. To this end, future work should examine the extent to which different neural responsivity may be moderated by the social valence of the stimuli; and whether these associations reflect broader, low-level auditory processing differences or if these associations are more specific to socially-relevant, human speech.

### 4.2. Limitations

While the inclusion of a broad range of neuroimaging modalities and techniques is a strength of our paper, one limitation is that the literature reviewed spans a range of experimental paradigms with varying stimuli and analytic approaches. This limits the extent to which we can draw strong conclusions across studies. As an example of this, only one study used nursery rhymes as their stimuli (Menn et al., 2022), which is more natural for an infant to listen to compared to isolated syllables. Indeed, it is possible that the lack of naturalness in the stimuli may constrain the generalizability of these findings to more naturalistic settings (Lalor et al., 2009; Lalor and Foxe, 2010; Ding and Simon, 2011). That is, neural responses to speech units (e.g., syllables) embedded in continuous, naturalistic speech (like a sung nursery rhyme) are different from responses to stimuli presented in isolation of greater linguistic context (e.g., as in single syllable presentation), even though the speech stimuli may be identical (Bonte et al., 2006).

Another limitation of this review is that we could only include articles published in English, limiting the inclusion of information that may have been published in other languages. In addition, our well-defined systematic review protocol omitted many articles due to constraints imposed by the protocol and inclusion criteria, which were set with the intent to elucidate neurobiological mechanisms underlying speech and language development exclusively in infants (0 to 12 months), during a critical time in development when formal language is emerging. Hence, the number of studies explored is limited. Nevertheless, this systematic review comprehensively presents the landscape of the extant literature on both structural and functional neurobiological differences in EL infants.

Finally, our review includes many studies that were conducted by the same group. For instance, of the fourteen EEG studies included in our review, 64% of those studies were conducted by the same research team in Boston, possibly limiting generalizability to populations residing in other geographic regions. This limitation highlights the fact that this is highly specialized work, often requiring extensive training and expensive equipment that tends to be limited to academic settings.

### 4.3. Future directions

Traditionally, studies of speech and language processing in LL and EL infants has utilized speech syllables presented in isolation. The use of more ecologically relevant stimuli, such as nursery rhymes (e.g., Attaheri et al., 2022), holds promise for answering questions regarding neural mechanisms underlying speech and language function and development in young children on the autism spectrum. In addition, machine learning-based approaches applied to neurophysiological or imaging data, such as support vector machine classifiers (as used in Peck et al., 2021 reviewed here) offer a potential data analytic technique to harmonize results across studies. Although the application of these algorithms to heterogeneous data has shown variable classification accuracy (ranging from 7 to 100% in Peck et al., 2021), there are methods for improvement (for a review see, Xie et al., 2019). For example, utilizing more sophisticated techniques for feature selection from EEG data could enhance the interpretability of the specific features used during classification. For example, spectral features of the speech stimuli could be pre-selected for neurophysiological speech decoding analyses (as in Yi et al., 2017).

As another future direction, prospective, longitudinal designs should begin to examine the feasibility and acceptability of remote EEG testing. Indeed, the last decade has seen an improvement in the size and portability of neurophysiological equipment (Niso et al., 2023), enabling researchers to reach remote populations and expand research opportunities to monitor neural deviations in early development.

## 5. Conclusion

This systematic review is the first to synthesize evidence across studies using various neuroimaging techniques to examine speech/language function and development in EL infants aged 0 to 12 months. Key findings suggest that EL infants may exhibit atypical language-related lateralization, alterations in structural and functional connectivity, and different patterns of neural sensitivity to speech or non-speech human vocalizations. Future research should aim to harmonize experimental paradigms both within and across neuroimaging modalities and additionally address the feasibility, acceptability, and scalability of implementing such methodologies in non-academic, community-based settings.

## Data availability statement

The raw data supporting the conclusions of this article will be made available by the authors, without undue reservation.

## Author contributions

JM, KS, RL, and RR conceptualized and designed the current study. JM and KS completed the search, data extraction, initial article review, and full-text review. JM, KS, and RR wrote the first draft of the manuscript. All authors revised, read, and approved the final submitted version.
