# Supplementary material for: Neural correlates and predictors of speech and language development in infants at elevated likelihood for autism: a systematic review
Source: Front Hum Neurosci. 2023 Aug 17;17:1211676. doi: 10.3389/fnhum.2023.1211676 (PMC10469683; doi:10.3389/fnhum.2023.1211676)
Supplement: Supplementary file 1 [file Table_1.docx]

| **Paper** | **Reason** |
| --- | --- |
| Bosl et al., 2018 | 4 |
| Cantiani et al., 2021 | 6 |
| Chenausky & Tager-Flusberg, 2017 | 3 |
| Cohen et al., 2013 | 5 |
| Damiano-Goodwin et al., 2018 | 6 |
| Darki et al., 2021 | 4 |
| Dean et al., 2020 | 2 |
| Denisova, 2019 | 4 |
| Denisova & Zhao, 2017 | 4 |
| Elison et al., 2013a | 4 |
| Elison et al., 2013b | 4 |
| Emerson et al., 2017 | 5 |
| Finch et al., 2018 | 6 |
| Fingher et al., 2017 | 5 |
| Girault et al., 2020 | 3 |
| Gabard-Durnam, 2019 | 4 |
| Glauser et al., 2022 | 4 |
| Godel et al., 2021 | 4 |
| Hazlett et al., 2012 | 4 |
| Hazlett et al., 2017 | 4 |
| Jones et al., 2017 | 4 |
| Keehn et al., 2015 | 4 |
| Kuhl et al., 2013 | 5 |
| Lewis et al., 2017 | 4 |
| Lombardo et al., 2021 | 5 |
| Lombardo et al., 2015 | 5 |
| McKinnon et al., 2019 | 4 |
| Nyström et al., 2021 | 4 |
| Orekhova, 2014 | 6 |
| Perdue et al., 2017 | 3 |
| Romeo et al., 2022 | 6 |
| Shen et al., 2017 | 4 |
| Simon et al., 2017 | 4 |
| St John et al., 2016 | 3 |
| Wilkinson et al., 2019 | 6 |
| Wolff et al., 2015 | 4 |
| Wolff et al., 2017 | 4 |

**Supplementary Table 1**. **Papers excluded after full-text review and reason for exclusion.**

*Note:* 1=Not English; 2= Not peer reviewed or original research; 3=No neural metric; 4=Neural metric included, but no speech or language measure; 5=No EL vs. LL group comparison; 6=Outside included age range

**Supplementary References**

Bosl, W. J., Tager-Flusberg, H., and Nelson, C. A. (2018). EEG Analytics for Early Detection of Autism Spectrum Disorder: A data-driven approach. *Sci Rep* 8, 6828. doi: [10.1038/s41598-018-24318-x](https://doi.org/10.1038/s41598-018-24318-x).

Cantiani, C., Riva, V., Dondena, C., Riboldi, E. M., Lorusso, M. L., and Molteni, M. (2021). Detection without further processing or processing without automatic detection? Differential ERP responses to lexical-semantic processing in toddlers at high clinical risk for autism and language disorder. *Cortex* 141, 465–481. doi: [10.1016/j.cortex.2021.04.020](https://doi.org/10.1016/j.cortex.2021.04.020).

Chenausky, K., and Tager-Flusberg, H. (2017). Acquisition of voice onset time in toddlers at high and low risk for autism spectrum disorder: VOT acquisition in toddlers at risk for ASD. *Autism Research* 10, 1269–1279. doi: [10.1002/aur.1775](https://doi.org/10.1002/aur.1775).

Cohen, I. L., Gardner, J. M., Karmel, B. Z., Phan, H. T. T., Kittler, P., Gomez, T. R., et al. (2013). Neonatal Brainstem Function and 4-Month Arousal-Modulated Attention Are Jointly Associated With Autism: Brainstem, arousal-modulated attention, and ASD. *Autism Res* 6, 11–22. doi: [10.1002/aur.1259](https://doi.org/10.1002/aur.1259).

Damiano-Goodwin, C. R., Woynaroski, T. G., Simon, D. M., Ibañez, L. V., Murias, M., Kirby, A., et al. (2018). Developmental sequelae and neurophysiologic substrates of sensory seeking in infant siblings of children with autism spectrum disorder. *Developmental Cognitive Neuroscience* 29, 41–53. doi: [10.1016/j.dcn.2017.08.005](https://doi.org/10.1016/j.dcn.2017.08.005).

Darki, F., Nyström, P., McAlonan, G., Bölte, S., and Falck-Ytter, T. (2021). T1-Weighted/T2-Weighted Ratio Mapping at 5 Months Captures Individual Differences in Behavioral Development and Differentiates Infants at Familial Risk for Autism from Controls. *Cerebral Cortex* 31, 4068–4077. doi: [10.1093/cercor/bhab069](https://doi.org/10.1093/cercor/bhab069).

Dean, D. C., Freeman, A., and Lainhart, J. (2020). The development of the social brain in baby siblings of children with autism: *Current Opinion in Psychiatry* 33, 110–116. doi: [10.1097/YCO.0000000000000572](https://doi.org/10.1097/YCO.0000000000000572).

Denisova, K. (2019). Failure to attune to language predicts autism in high risk infants. *Brain and Language* 194, 109–120. doi: [10.1016/j.bandl.2019.04.002](https://doi.org/10.1016/j.bandl.2019.04.002).

Denisova, K., and Zhao, G. (2017). Inflexible neurobiological signatures precede atypical development in infants at high risk for autism. *Sci Rep* 7, 11285. doi: [10.1038/s41598-017-09028-0](https://doi.org/10.1038/s41598-017-09028-0).

Elison, J. T., Paterson, S. J., Wolff, J. J., Reznick, J. S., Sasson, N. J., Gu, H., et al. (2013a). White Matter Microstructure and Atypical Visual Orienting in 7-Month-Olds at Risk for Autism. *AJP* 170, 899–908. doi: [10.1176/appi.ajp.2012.12091150](https://doi.org/10.1176/appi.ajp.2012.12091150).

Elison, J. T., Wolff, J. J., Heimer, D. C., Paterson, S. J., Gu, H., Hazlett, H. C., et al. (2013b). Frontolimbic neural circuitry at 6 months predicts individual differences in joint attention at 9 months. *Dev Sci* 16, 186–197. doi: [10.1111/desc.12015](https://doi.org/10.1111/desc.12015).

Emerson, R. W., Adams, C., Nishino, T., Hazlett, H. C., Wolff, J. J., Zwaigenbaum, L., et al. (2017). Functional neuroimaging of high-risk 6-month-old infants predicts a diagnosis of autism at 24 months of age. *Sci. Transl. Med.* 9, eaag2882. doi: [10.1126/scitranslmed.aag2882](https://doi.org/10.1126/scitranslmed.aag2882).

Finch, K. H., Tager‐Flusberg, H., and Nelson, C. A. (2018). Neural responses to linguistic stimuli in children with and without autism spectrum disorder. *Eur J Neurosci* 47, 709–719. doi: [10.1111/ejn.13721](https://doi.org/10.1111/ejn.13721).

Fingher, N., Dinstein, I., Ben-Shachar, M., Haar, S., Dale, A. M., Eyler, L., et al. (2017). Toddlers later diagnosed with autism exhibit multiple structural abnormalities in temporal corpus callosum fibers. *Cortex* 97, 291–305. doi: [10.1016/j.cortex.2016.12.024](https://doi.org/10.1016/j.cortex.2016.12.024).

Girault, J. B., Swanson, M. R., Meera, S. S., Grzadzinski, R. L., Shen, M. D., et al. (2020). Quantitative trait variation in ASD probands and toddler sibling outcomes at 24 months. *J Neurodevelop Disord* 12, 5. doi: [10.1186/s11689-020-9308-7](https://doi.org/10.1186/s11689-020-9308-7).

Gabard-Durnam, L. J., Wilkinson, C., Kapur, K., Tager-Flusberg, H., Levin, A. R., and Nelson, C. A. (2019). Longitudinal EEG power in the first postnatal year differentiates autism outcomes. *Nat Commun* 10, 4188. doi: [10.1038/s41467-019-12202-9](https://doi.org/10.1038/s41467-019-12202-9).

Glauser, J., Wilkinson, C. L., Gabard-Durnam, L. J., Choi, B., Tager-Flusberg, H., and Nelson, C. A. (2022). Neural correlates of face processing associated with development of social communication in 12-month infants with familial risk of autism spectrum disorder. *J Neurodevelop Disord* 14, 6. doi: [10.1186/s11689-021-09413-x](https://doi.org/10.1186/s11689-021-09413-x).

Godel, M., Andrews, D. S., Amaral, D. G., Ozonoff, S., Young, G. S., Lee, J. K., et al. (2021). Altered Gray-White Matter Boundary Contrast in Toddlers at Risk for Autism Relates to Later Diagnosis of Autism Spectrum Disorder. *Front. Neurosci.* 15, 669194. doi: [10.3389/fnins.2021.669194](https://doi.org/10.3389/fnins.2021.669194).

Hazlett, H. C., Gu, H., McKinstry, R. C., Shaw, D. W. W., Botteron, K. N., Dager, S. R., et al. (2012). Brain Volume Findings in 6-Month-Old Infants at High Familial Risk for Autism. *AJP* 169, 601–608. doi: [10.1176/appi.ajp.2012.11091425](https://doi.org/10.1176/appi.ajp.2012.11091425).

Hazlett, H. C., Gu, H., Munsell, B. C., Kim, S. H., Styner, M., et al. (2017). Early brain development in infants at high risk for autism spectrum disorder. *Nature* 542, 348–351. doi: [10.1038/nature21369](https://doi.org/10.1038/nature21369).

Jones, E. J. H., Dawson, G., Kelly, J., Estes, A., and Webb, S. J. (2017). Parent-delivered early intervention in infants at risk for ASD: Effects on electrophysiological and habituation measures of social attention: Intervention in infants at risk for ASD. *Autism Research* 10, 961–972. doi: [10.1002/aur.1754](https://doi.org/10.1002/aur.1754).

Keehn, B., Vogel-Farley, V., Tager-Flusberg, H., and Nelson, C. A. (2015). Atypical Hemispheric Specialization for Faces in Infants at Risk for Autism Spectrum Disorder: Atypical lateralization in high-risk infants. *Autism Res* 8, 187–198. doi: [10.1002/aur.1438](https://doi.org/10.1002/aur.1438).

Kuhl, P. K., Coffey-Corina, S., Padden, D., Munson, J., Estes, A., and Dawson, G. (2013). Brain Responses to Words in 2-Year-Olds with Autism Predict Developmental Outcomes at Age 6. *PLoS ONE* 8, e64967. doi: [10.1371/journal.pone.0064967](https://doi.org/10.1371/journal.pone.0064967).

Lewis, J. D., Evans, A. C., Pruett, J. R., Botteron, K. N., McKinstry, R. C., Zwaigenbaum, L., et al. (2017). The Emergence of Network Inefficiencies in Infants With Autism Spectrum Disorder. *Biological Psychiatry* 82, 176–185. doi: [10.1016/j.biopsych.2017.03.006](https://doi.org/10.1016/j.biopsych.2017.03.006).

Lombardo, M. V., Eyler, L., Pramparo, T., Gazestani, V. H., Hagler, D. J., Chen, C.-H., et al. (2021). Atypical genomic cortical patterning in autism with poor early language outcome. *Sci. Adv.* 7, eabh1663. doi: [10.1126/sciadv.abh1663](https://doi.org/10.1126/sciadv.abh1663).

Lombardo, M. V., Pierce, K., Eyler, L. T., Carter Barnes, C., Ahrens-Barbeau, C., Solso, S., et al. (2015). Different Functional Neural Substrates for Good and Poor Language Outcome in Autism. *Neuron* 86, 567–577. doi: [10.1016/j.neuron.2015.03.023](https://doi.org/10.1016/j.neuron.2015.03.023).

McKinnon, C. J., Eggebrecht, A. T., Todorov, A., Wolff, J. J., Elison, J. T., Adams, C. M., et al. (2019). Restricted and Repetitive Behavior and Brain Functional Connectivity in Infants at Risk for Developing Autism Spectrum Disorder. *Biological Psychiatry: Cognitive Neuroscience and Neuroimaging* 4, 50–61. doi: [10.1016/j.bpsc.2018.09.008](https://doi.org/10.1016/j.bpsc.2018.09.008).

Nyström, P., Jones, E., Darki, F., Bölte, S., and Falck-Ytter, T. (2021). Atypical Topographical Organization of Global Form and Motion Processing in 5-Month-Old Infants at Risk for Autism. *J Autism Dev Disord* 51, 364–370. doi: [10.1007/s10803-020-04523-2](https://doi.org/10.1007/s10803-020-04523-2).

Orekhova, E. V., Elsabbagh, M., Jones, E. J., Dawson, G., Charman, T., et al. (2014). EEG hyper-connectivity in high-risk infants is associated with later autism. *J Neurodevelop Disord* 6, 40. doi: [10.1186/1866-1955-6-40](https://doi.org/10.1186/1866-1955-6-40).

Perdue, K. L., Edwards, L. A., Tager-Flusberg, H., and Nelson, C. A. (2017). Differing Developmental Trajectories in Heart Rate Responses to Speech Stimuli in Infants at High and Low Risk for Autism Spectrum Disorder. *J Autism Dev Disord* 47, 2434–2442. doi: [10.1007/s10803-017-3167-4](https://doi.org/10.1007/s10803-017-3167-4).

Romeo, R. R., Choi, B., Gabard-Durnam, L. J., Wilkinson, C. L., Levin, A. R., Rowe, M. L., et al. (2022). Parental Language Input Predicts Neuroscillatory Patterns Associated with Language Development in Toddlers at Risk of Autism. *J Autism Dev Disord* 52, 2717–2731. doi: [10.1007/s10803-021-05024-6](https://doi.org/10.1007/s10803-021-05024-6).

Shen, M. D., Kim, S. H., McKinstry, R. C., Gu, H., Hazlett, H. C., Nordahl, C. W., et al. (2017). Increased Extra-axial Cerebrospinal Fluid in High-Risk Infants Who Later Develop Autism. *Biological Psychiatry* 82, 186–193. doi: [10.1016/j.biopsych.2017.02.1095](https://doi.org/10.1016/j.biopsych.2017.02.1095).

Simon, D. M., Damiano, C. R., Woynaroski, T. G., Ibañez, L. V., Murias, M., Stone, W. L., et al. (2017). Neural Correlates of Sensory Hyporesponsiveness in Toddlers at High Risk for Autism Spectrum Disorder. *J Autism Dev Disord* 47, 2710–2722. doi: [10.1007/s10803-017-3191-4](https://doi.org/10.1007/s10803-017-3191-4).

St. John, T., Estes, A. M., Dager, S. R., Kostopoulos, P., Wolff, J. J., Pandey, J., et al. (2016). Emerging Executive Functioning and Motor Development in Infants at High and Low Risk for Autism Spectrum Disorder. *Front. Psychol.* 7. doi: [10.3389/fpsyg.2016.01016](https://doi.org/10.3389/fpsyg.2016.01016).

Wilkinson, C. L., Levin, A. R., Gabard‐Durnam, L. J., Tager‐Flusberg, H., and Nelson, C. A. (2019). Reduced frontal gamma power at 24 months is associated with better expressive language in toddlers at risk for autism. *Autism Research* 12, 1211–1224. doi: [10.1002/aur.2131](https://doi.org/10.1002/aur.2131).

Wolff, J. J., Gerig, G., Lewis, J. D., Soda, T., Styner, M. A., Vachet, C., et al. (2015). Altered corpus callosum morphology associated with autism over the first 2 years of life. *Brain* 138, 2046–2058. doi: [10.1093/brain/awv118](https://doi.org/10.1093/brain/awv118).

Wolff, J. J., Swanson, M. R., Elison, J. T., Gerig, G., Pruett, J. R., et al. (2017). Neural circuitry at age 6 months associated with later repetitive behavior and sensory responsiveness in autism. *Molecular Autism* 8, 8. doi: [10.1186/s13229-017-0126-z](https://doi.org/10.1186/s13229-017-0126-z).
